# Sirt6 is required for spermatogenesis in mice

**DOI:** 10.18632/aging.103641

**Published:** 2020-09-11

**Authors:** Huafang Wei, Muhammad Babar Khawar, Wenhao Tang, Lina Wang, Liying Wang, Chao Liu, Hui Jiang, Wei Li

**Affiliations:** 1 State Key Laboratory of Stem Cell and Reproductive Biology, Institute of Zoology, Chinese Academy of Sciences, Beijing 100101, China; 2 University of the Chinese Academy of Sciences, Beijing 100049, China; 3 Department of Urology, Peking University Third Hospital, Beijing 100191, China; 4 Department of Andrology, Peking University Third Hospital, Beijing 100191, China; 5 Department of Reproductive Medicine Center, Peking University Third Hospital, Beijing 100191, China; 6 Department of Human Sperm Bank, Peking University Third Hospital, Beijing 100191, China

**Keywords:** Sirt6, spermatogenesis, elongated spermatid arrest, infertility

## Abstract

SIRT6, a nuclear protein, has been implicated in a number of essential cellular processes, such as the DNA damage response, metabolic homeostasis, inflammation, tumorigenesis and aging. However, the role of Sirt6 in the regulation of spermatogenesis is yet unknown. In the present study, we successfully generated *Sirt6^-/-^* mice on a C57BL6/ICR mixed background and found that some *Sirt6^-/-^* mice survived beyond eight weeks. We further revealed that spermatogenesis in *Sirt6^-/-^* mice was arrested at the elongated spermatid stage. *Sirt6^-/-^* male mice were completely infertile and had an increased number of apoptotic spermatids. To our surprise, deacetylation activities of SIRT6 on H3K9ac, H3K18ac and H3K56c were not required for spermatogenesis. Therefore, our findings establish a novel link between Sirt6 and male fertility, suggesting an essential role of Sirt6 in spermatogenesis.

## INTRODUCTION

Spermatogenesis is a complicated and highly ordered process of male gamete production that is characterized by massive cellular renovation and differentiation. Spermatogenesis is comprised of self-renewal and differentiation of spermatogonia, meiosis of spermatocytes and spermiogenesis of spermatids [1]. After a single round of DNA replication followed by two successive divisions, the diploid spermatocytes produce four haploid spermatids. Compared with mitosis, some unique events occur during meiosis, such as programmed double-strand DNA breaks, meiotic recombination, synapsis and crossover formation, that if defective usually results in meiotic arrest and ultimately infertility [2]. Subsequently, during spermiogenesis, the differentiation process of spermatids occurs from the end of meiosis to the release of the mature spermatozoa. During this time, spermatids undergo several structural reorganizations, including the formation of the acrosome, the condensation of the nuclear chromatin, the rearrangement of the mitochondria, the assembly of the sperm flagella, and the removal of unnecessary cytoplasm to facilitate motility of spermatozoa [3, 4]. Failures in any of these crucial events result in abnormalities in the sperm’s morphology and function [5]. However, the molecular mechanism underlying spermatid differentiation is a very complex process and remains poorly understood.

Silent information regulator 2 (Sir2) is an evolutionarily conserved NAD^+^-dependent histone deacetylase [6]. Seven sirtuin family proteins (Sirt1-7) have been identified in mammals, they are localized in different subcellular compartments, and have been identified as Sir2 orthologs. [7–9]. SIRT1 and SIRT6 are predominately located within the nucleus [10], whereas SIRT7 is located within the nucleolus. SIRT2 is predominantly found in the cytoplasm [11, 12], whereas SIRT3, SIRT4 and SIRT5 are localized in mitochondria [13]. Together, they respond to metabolic challenges, inflammatory signals or hypoxic/ oxidative stress, and are associated with aging and longevity [14, 15]. The roles of sirtuins in the regulation of fertility were noticed in 2003 when a defective reproductive phenotype was observed in male and female *Sirt1^-/-^* mice [16]. Accumulating evidence showed that Sirt1 is associated with ovarian reserve, granulosa cells proliferation and survival [17–19]. In addition, SIRT1, SIRT2, SIRT3 and SIRT6 were found to improve the competence of oocyte growth and maturation [20–23]. Recently, SIRT1, SIRT2 and SIRT3 have emerged as protectors of oocytes against postovulatory aging [24]. As for the male, SIRT1 is found to be involved in spermatogenesis by influencing multiple processes of both somatic and germ cells in the testis [25–27]. Although some of the functional roles of Sirtuin family members have been uncovered, many aspects still need further exploration.

Sirt6 is one of the relatively less studied Sirtuin family members that is involved in the reproductive process, and plays multiple roles in homeostasis, lifespan, and disease [28–30]. Similar to *Sirt1^-/-^* C57BL6 mice, *Sirt6^-/-^* C57BL6 mice are slow growing at 2–3 weeks, develop abnormalities that include profound lymphopenia, loss of subcutaneous fat, lordokyphosis, severe metabolic defects, and eventually die at the age of about 4 weeks [31]. Male mice fed with a high-fat diet had impaired fertility due to obesity, along with significantly decreased SIRT6 protein and increased acetylated H3K9 and DNA damage in the nucleus [32], suggesting that Sirt6 might be involved in spermatogenesis.

To investigate whether Sirt6 plays an essential role in spermatogenesis, we generated mixed background *Sirt6^-/-^* mice and found that some *Sirt6^-/-^* mice survived beyond eight weeks. The spermatogenesis in *Sirt6^-/-^* mice was arrested at the elongated spermatid stage with increased apoptotic spermatids. To our surprise, deacetylation activities of SIRT6 on H3K9ac, H3K18ac and H3K56c are not required for spermatogenesis, which have been shown to have a significant impact in somatic cells. Therefore, our findings establish a novel link between Sirt6 and male fertility, suggesting an essential role of Sirt6 in spermatogenesis.

## RESULTS

### Sirt6 is required for spermatogenesis

**Figure 1:**
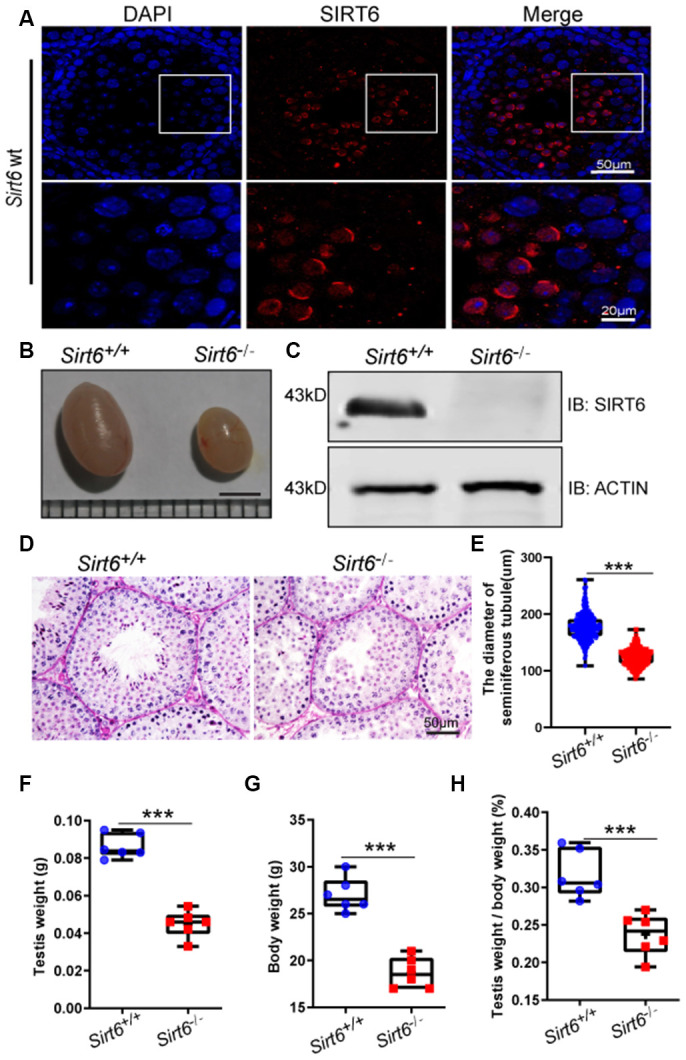
**SIRT6 protein expression and localization in mice testes.** (**A**) Testicular sections of *Sirt6^+/+^* stained for SIRT6 (red) and DAPI (blue). SIRT6 is localized in the spermatids. 8-week mice, n=3. (**B**) The testes of *Sirt6^-/-^* mice were smaller than those of the *Sirt6^+/+^* mice. 8-week mice, n=6, scale bar=3 mm. (**C**) SIRT6 protein levels were dramatically reduced in the testes of the *Sirt6^-/-^* mice. ACTIN was used as the loading control. 8-week mice, n=3. (**D**) Histological analysis of *Sirt6^+/+^* and *Sirt6^-/-^* mice seminiferous tubules by PAS-hematoxylin staining. 8-week mice, n=4. (**E**) The diameter of the seminiferous tubules in *Sirt6^-/-^* mice was smaller than that in control mice. *Sirt6^+/+^*, 177.43±2.36μm; *Sirt6^-/-^*, 123.94±1.92μm. 8-week mice, n=6; 300 seminiferous tubules were used for each group. Data are presented as mean ± SEM. ***P < 0.001. (**F**) Quantification of testis weight of the *Sirt6^+/+^* and *Sirt6^-/-^* mice. The testis weight of *Sirt6^-/-^* mice was significantly reduced. *Sirt6^+/+^*, 8.60±1.30% g; *Sirt6^-/-^*, 4.49±1.40% g. 8-week mice, n=6. Data are presented as mean ± SEM. ***P < 0.001. (**G**) Quantification of body weight of the *Sirt6^+/+^* and *Sirt6^-/-^* mice. The body weight of *Sirt6^-/-^* mice was reduced. *Sirt6^+/+^*, 27.00±0.55 g; *Sirt6^-/-^*, 18.67±0.30g. 8-week mice, n=6. Data are presented as mean ± SEM. ***P < 0.001. (**H**) The testis weight/ body weight of *Sirt6^-/-^* mice was also reduced. *Sirt6^+/+^*, 0.32±0.07%; *Sirt6^-/-^*, 0.04±0.07%. 8-week mice, n=6. Data are presented as mean ± SEM. ***P < 0.001.

To study the potential role of Sirt6 in the testes, we first carried out immunostaining to investigate the location of SIRT6 in the seminiferous tubules of mice. We found that SIRT6 was specifically expressed in round spermatids and elongated spermatids (Figure 1A). Next, we generated Sirt6 global-knockout (hereafter called *Sirt6^-/-^*) mice on a C57BL6/ICR mixed background. Unlike previous reports [31], we found that most of the *Sirt6^-/-^* mice died within 4 weeks of age, while some *Sirt6^-/-^* mice survived beyond 8 weeks. With the surviving *Sirt6^-/-^* mice, we quantified pup numbers from 146 pregnant mice and identified the genotype of pups in each cage within a week of birth. Mating of *Sirt6^+/-^* and *Sirt6^+/-^* mice resulted in a mean litter size of about 9 pups, and gender distribution (699:751) was roughly consistent with Mendelian inheritance. The survival rate was about 4.13% from 218 *Sirt6*-knockout pups. We then performed western blot analysis to determine the expression levels of SIRT6 in the testes, and found that SIRT6 was significantly decreased in *Sirt6^-/-^* testes compared to *Sirt6^+/+^* testes (Figure 1C). We characterized the 8 week-old knockout mice and found that they had lower body weight and were smaller than that of the *Sirt6^+/+^* littermates (Figure 1G). The testis size and weight were also significantly decreased compared with that of *Sirt6^+/+^* mice (Figure 1B, 1F and 1H). Further histological examination showed that *Sirt6^-/-^* testes contained few elongated spermatids (Figure 1D), and in the cross-section of *Sirt6^+/+^* and *Sirt6^-/-^* testes, we choose 300 seminiferous tubules of different sizes and determined by microscopy that the mean diameter of seminiferous tubules of *Sirt6^-/-^* mice was significantly decreased compared with that in the control group (Figure 1E). Together, the loss of elongated spermatids and smaller testes in *Sirt6^-/-^* mice suggest that Sirt6 deficiency severely impairs spermatogenesis.

### Sirt6 is dispensable to meiosis during spermatogenesis

**Figure 2:**
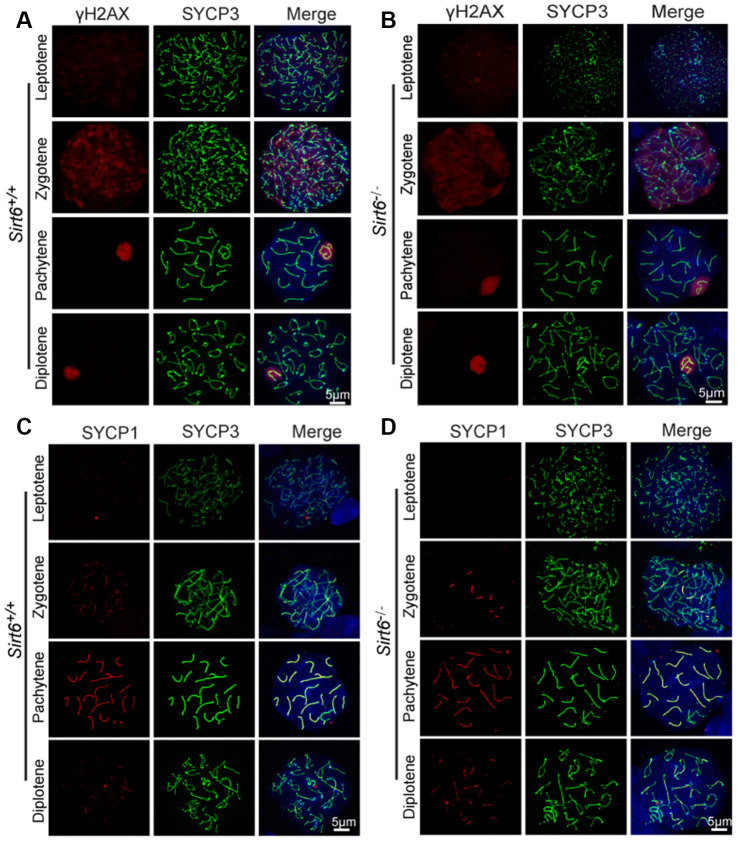
**Sirt6 is not essential to meiosis during spermatogenesis.** (**A**, **B**) Spermatocytes of *Sirt6^+/+^* and *Sirt6^-/-^* stained with SYCP3 (green) and γH2AX (red) antibodies, 8-week mice, n=4; 200 cells were used for each group. (**C**, **D**) Spermatocytes of *Sirt6^+/+^* and *Sirt6^-/-^* stained for SYCP3 (green) and SYCP1 (red), 8-week mice, n=4; 200 cells were used for each group.

Chromosomal synapsis depends on the formation of the synaptonemal complex, which is comprised of three main proteins: SYCP1, SYCP2, and SYCP3 [33]. SYCP1 is the central element that links two homologous chromosomes for pairing and helps in the formation of the chiasmata in the pachytene stage. SYCP3 and SYCP2 are the lateral elements of the synaptonemal complex and are distributed along the chromosome axis [33]. The phosphorylation of histone H2AX at serine 139 (termed γH2AX) is essential for the production of programmed DNA double-strand breaks (DSBs) which are required for the initiation of meiotic recombination [34]. Concomitant with DSB formation and repair, γH2AX accumulates in the nucleus during the leptotene (distributed globally) and zygotene stages, decreases in the autosomes and concentrates only in the sex chromosomes during the pachytene stage. To investigate the mechanism underlying the infertility of *Sirt6^-/-^* mice, we first examined meiotic progression by immunostaining the axial components of the synaptonemal complex and γH2Ax. Immunostaining of SYCP3 and γH2Ax in spermatocytes revealed that the initiation of DSB formation appeared starting in the leptotene stage and remained until the pachytene stage, and DNA damage was repaired in all autosomes and concentrated only in the sex chromosomes in both *Sirt6^+/+^* and *Sirt6^-/-^* mice (Figure 2A and 2B), suggesting that Sirt6 is not essential for DSB repair in meiosis. We then examined homolog synapses via immunostaining of the synaptonemal complex protein SYCP1 that loads only to the synapsed chromosomes [35]. SYCP1 was found to be colocalized with SYCP3 (except on XY chromosomes) in most of the pachytene spermatocytes (Figure 2C and 2D), indicating that full synapsis was acquired in the spermatocytes of both *Sirt6^+/+^* and *Sirt6^-/-^* mice. On the basis of these findings, we concluded that Sirt6 is not essential to the meiotic progression of germ cells during spermatogenesis. Thus, it might be the subsequent spermiogenesis stages that were affected in *Sirt6^-/-^* mice.

### Loss of Sirt6 results in spermatogenic arrest at the elongated spermatid stage

**Figure 3:**
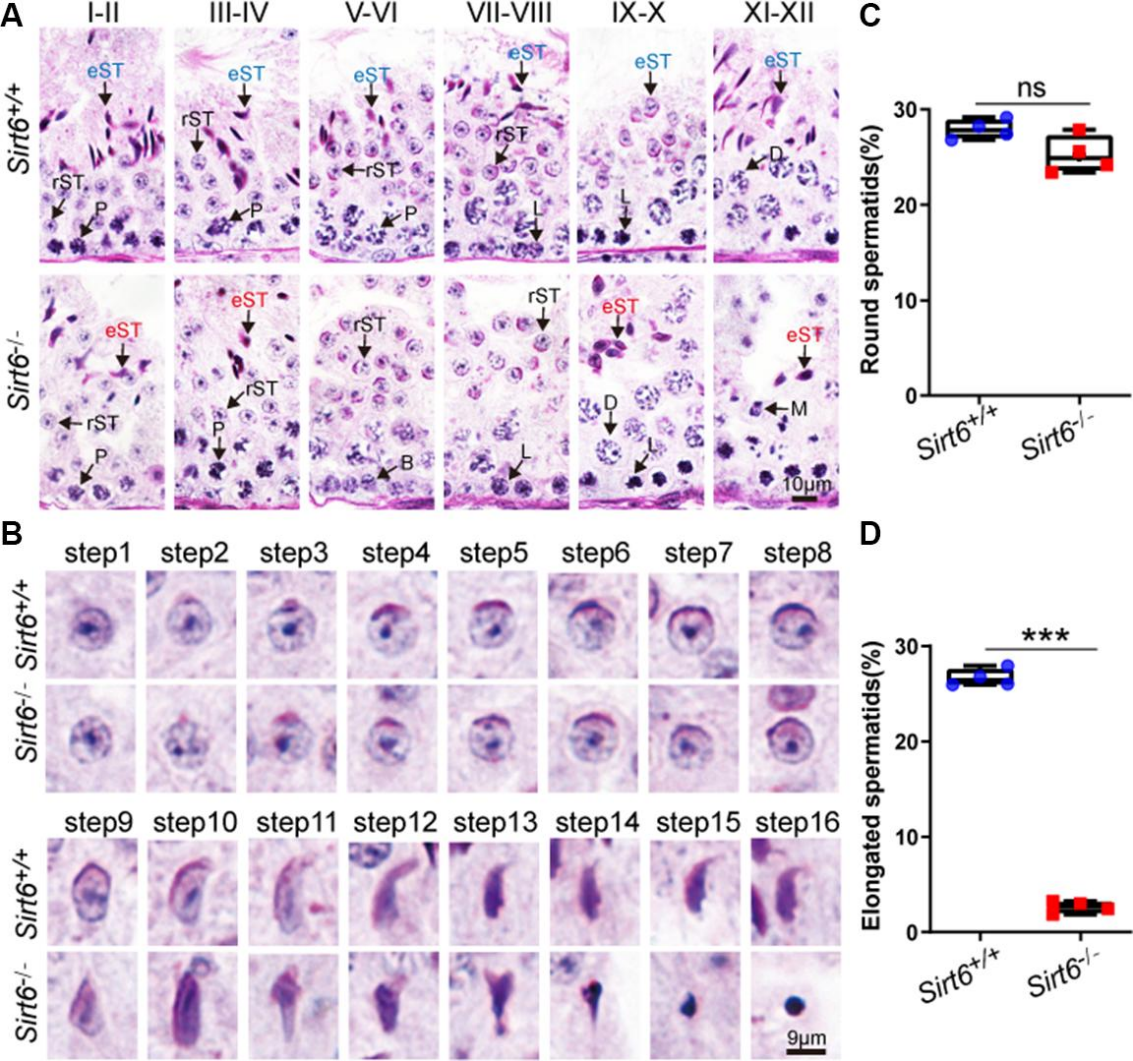
***Sirt6* deficiency leads to spermatogenic arrest at the elongated spermatid stage.** (**A**) Paraffin-embedded sections of seminiferous tubules and testes from *Sirt6^+/+^* and *Sirt6^-/-^* were stained with PAS-hematoxylin. B, B type spermatogonial stem cells; L, leptotene spermatocytes; D, diplotene spermatocytes; P, pachytene spermatocytes; rST, round spermatids; eST, elongated spermatids; M, meiotic divisions. 8-week mice, n=4. (**B**) Different developmental steps of single round spermatid and elongated spermatids from *Sirt6^+/+^* and *Sirt6^-/-^* testes. 8-week mice, n=4. (**C**) Quantification of the proportion of round spermatids in the *Sirt6^+/+^* and *Sirt6^-/-^* mice. *Sirt6^+/+^*, 27.91±1.03%; *Sirt6^-/-^*, 25.27±1.96%. 8-week mice, n=4; 96 seminiferous tubules were used for each group. (**D**) Quantification of the proportion of elongated spermatids in the *Sirt6^+/+^* and *Sirt6^-/-^* mice. *Sirt6^+/+^*, 26.67±0.48%; *Sirt6^-/-^*, 2.64±0.12%. 8-week mice, n=4; 96 seminiferous tubules were used for each group. Data are presented as mean ± SEM. ***P < 0.001.

The spermatogenesis process in mice is usually divided into 12 stages to describe germ cell development, which was characterized in tissue stained with Periodic Acid Schiff (PAS) and hematoxylin [36]. To determine exactly which spermatogenic stage was affected by SIRT6 deficiency, we performed PAS and hematoxylin staining and observed that spermatogonia, spermatocytes and round spermatids had a significantly reduced number of elongated spermatids in *Sirt6^-/-^* testes (Figure 3A), suggesting elongated spermatids development was disrupted in *Sirt6^-/-^* mice.

Moreover, the differentiation of haploid spermatids can be divided into 16 steps, and step-by-step spermatid differentiation can also be characterized with PAS and hematoxylin staining. The morphology of round spermatids in both *Sirt6^+/+^* and *Sirt6^-/-^* testes was fully developed and consisted of a well-assembled acrosome and nucleus (Figure 3B). During spermiogenesis, the round-shaped nucleus undergoes elongation and becomes falciform. These sperm-specific nuclear structures and shapes are considered to contribute to the generation of a more hydrodynamic sperm head that can quickly transit through the female reproductive tract and protects the genetic material from physical and chemical damages [37]. In the elongated spermatids of *Sirt6^+/+^* mouse, the acrosome was found to stop growing and was observed to change its morphology during steps 9-16. After a series of highly complex morphological changes, such as spermatid elongation and cytoplasm removal, spermiogenesis eventually results in producing mature sperm. The spermiogenesis process was considered fully normal in *Sirt6^+/+^* mice as indicated by mature sperm found in the testes. However, in *Sirt6^-/-^* mice, many of the spermatids were observed to have irregularly shaped heads (Figure 3B). Next, we quantified the proportion of round spermatids in both *Sirt6^+/+^* and *Sirt6^-/-^* testes, and found that the round spermatid developmental status of *Sirt6^-/-^* mice is consistent with that of *Sirt6^+/+^* mice (Figure 3C). However, the proportion of elongated spermatids was decreased significantly in *Sirt6^-/-^* testes (Figure 3D). Together, these findings indicate that SIRT6 deficiency blocks the spermiogenesis process, and loss of Sirt6 results in spermatogenic arrest at the elongated spermatid stage.

### SIRT6-deficiency leads to malformed spermatids with impaired acrosomes

**Figure 4:**
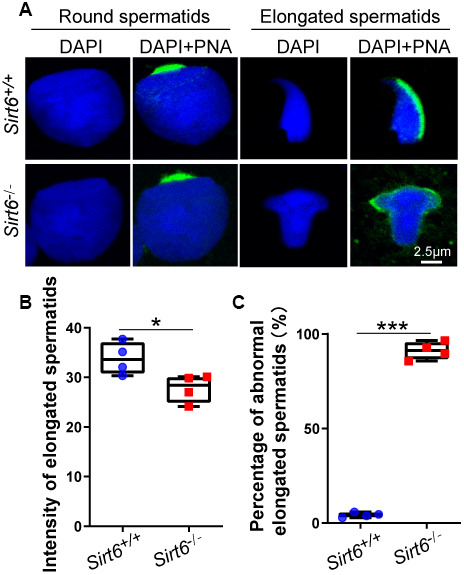
**SIRT6-deficiency leads to malformed spermatozoa with impaired acrosomes.** (**A**) Immunofluorescence staining of PNA (green) and DAPI (blue) in *Sirt6^+/+^* and *Sirt6^-/-^* spermatids. The acrosome is disrupted in *Sirt6^-/-^* spermatids. (**B**) Quantification of intensity of elongated spermatids in (**A**). *Sirt6^+/+^*, 33.84±0.90; *Sirt6^-/-^*, 27.79±0.84, 8-week mice, n=4; 150 cells were used for each group. Data are presented as mean ± SEM. *P < 0.05. (**C**) Quantification of disrupted elongated spermatids of the *Sirt6^+/+^* and *Sirt6^-/-^* mice. *Sirt6^+/+^*, 4.22±0.57%; *Sirt6^-/-^*, 91.16±1.08%, 8-week mice, n=4; 200 cells were used for each group. Data are presented as mean ± SEM. ***P < 0.001.

The acrosome is a unique membranous organelle located at the anterior portion of the sperm nucleus. The organelle plays an important role in the dispersion of cumulus cells and/or sperm penetration of the zona pellucida of the oocyte during fertilization [38]. Acrosome biogenesis consists of the following phases: the Golgi phase with round spermatids from step 1 to step 3 in the seminiferous tubules; the Cap phase with round spermatids from step 4 to step 8 in the seminiferous tubules; and the acrosome and maturation phases with elongated spermatids from stage step 9 to step 16 in the seminiferous tubules [39]. To test whether *Sirt6* knockout has any impact on acrosomes biogenesis, single-sperm immunofluorescence was performed. The acrosomes of round spermatids from both *Sirt6^+/+^* and *Sirt6^-/-^* mice were found to be well-developed while acrosomes of elongate spermatids from *Sirt6^-/-^* mice showed severe defects compared to *Sirt6^+/+^* mice (Figure 4A). Moreover, the acrosome signal of elongated spermatids of *Sirt6^-/-^* mice showed weaker fluorescence intensity than that of *Sirt6^+/+^* mice (Figure 4B), indicating acrosome biogenesis is affected in *Sirt6^-/-^* mice. Altogether, more than 90% of elongated spermatids were abnormal in the *Sirt6^-/-^* mice compared with 4.22% in the *Sirt6^+/+^* mice (Figure 4C), suggesting elongated spermatids are disrupted severely in *Sirt6^-/-^* mice. These results indicate that SIRT6-deficiency leads to malformed elongated spermatids with impaired acrosomes.

### *Sirt6* deficiency results in increased apoptotic spermatids

**Figure 5:**
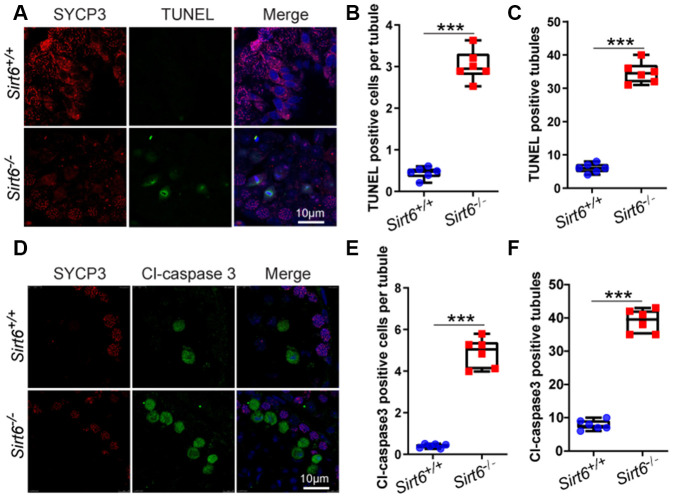
***Sirt6* deficiency results in increased apoptotic spermatids.** (**A**) Paraffin embedded sections from *Sirt6^+/+^* and *Sirt6^-/-^* testes were stained with TUNEL kit (green) and SYCP3 (red) to determine the presence of apoptotic cells. 8-week mice, n=6. (**B**) Quantification of TUNEL positive cells per tubule of the *Sirt6^+/+^* and *Sirt6^-/-^* mice. *Sirt6^+/+^*, 0.45±0.1; *Sirt6^-/-^*, 3.03±0.25. 8-week mice, n=6; 533 cells were used for each group. Data are presented as mean ± SEM. ***P < 0.001. (**C**) Quantification of TUNEL positive tubules of the *Sirt6^+/+^* and *Sirt6^-/-^* mice. *Sirt6^+/+^*, 6.00±0.49; *Sirt6^-/-^*, 34.67±0.53. 8-week mice, n=6; 228 seminiferous tubules were used for each group. Data are presented as mean ± SEM. ***P < 0.001. (**D**) Paraffin embedded sections from *Sirt6^+/+^* and *Sirt6^-/-^* testes were stained with Cl-caspase3 (green), SYCP3 (red) and DAPI (blue) to determine the presence of apoptotic cells. 8-week mice, n=6. (**E**) Quantification of Cl-caspase3 positive cells per tubule of the *Sirt6^+/+^* and *Sirt6^-/-^* mice. *Sirt6^+/+^*, 0.40±0.13; *Sirt6^-/-^*, 4.88±0.34. 8-week mice, n=6; 497cells were used for each group. Data are presented as mean ± SEM. ***P < 0.001. (**F**) Quantification of Cl-caspase3 positive tubules of the *Sirt6^+/+^* and *Sirt6^-/-^* mice. *Sirt6^+/+^*, 7.83±0.50; *Sirt6^-/-^*, 39.00±0.59. 8-week mice, n=6; 201 seminiferous tubules were used for each group. Data are presented as mean ± SEM. ***P < 0.001.

A large number of degenerated cells with highly condensed nuclei were found in the seminiferous epithelium of *Sirt6^-/-^* mice (Figure 3B, from step 9 to step 16). It is likely that these elongated spermatids underwent cell death. To confirm this possibility, we performed immunofluorescence staining via the terminal deoxynucleotidyl transferase dUTP nick end-labeling (TUNEL) assay and found that TUNEL positive cells per tubule and TUNEL positive tubules were increased in *Sirt6^-/-^* spermatids (Figure 5A–5C). To confirm this result, we performed cleaved caspase-3 staining to label apoptotic cells**,** which showed that *Sirt6* deletion significantly elevated the number of apoptotic spermatids (Figure 5D–5F). These results suggest that elongated spermatids may be lost due to cell death in *Sirt6^-/-^* mice.

### Histone deacetylation activities of SIRT6 might not be required for spermatogenesis

**Figure 6:**
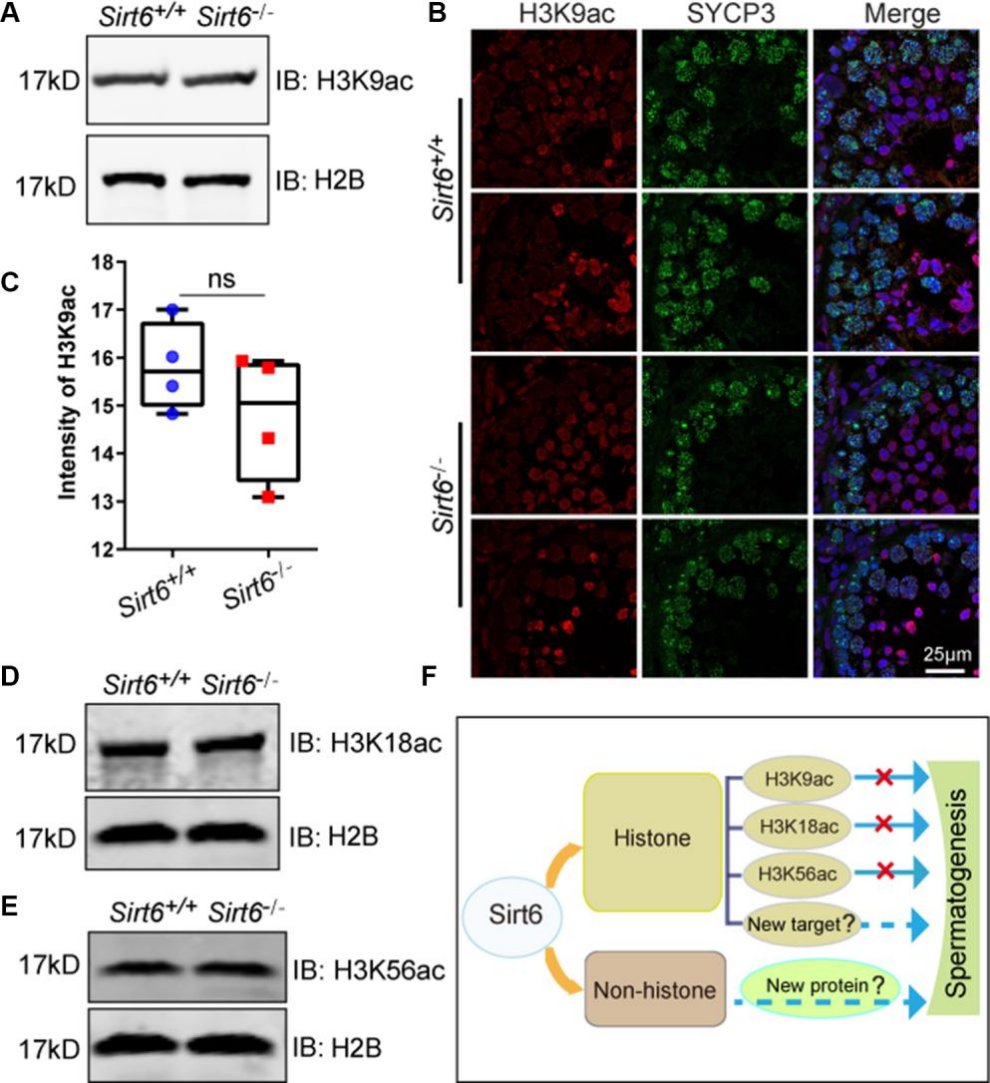
**Deacetylation activity of SIRT6 on H3K9ac, H3K18ac and H3K56c is not required for spermatogenesis.** (**A**) The levels of H3K9ac in *Sirt6^+/+^* and *Sirt6^-/-^* testes were detected by western blot. 8-week mice, n=3. (**B**) Localization of H3K9ac in *Sirt6^+/+^* and *Sirt6^-/-^* seminiferous tubules. Testes sections of *Sirt6^+/+^* and *Sirt6^-/-^* stained with SYCP3 (green) and H3K9ac (red) antibodies. 8-week mice, n=4. (**C**) Quantification of H3K9ac intensity of round spermatids and elongated spermatids in the *Sirt6^+/+^* and *Sirt6^-/-^* mice. *Sirt6^+/+^*, 15.82±0.48; *Sirt6^-/-^*, 14.78±0.58. 8-week mice, n=4; 200 cells were used for each group. Data are presented as mean ± SEM. (**D**) The levels of H3K18ac in *Sirt6^+/+^* and *Sirt6^-/-^* testes were detected by western blot. 8-week mice, n=3. (**E**) The levels of H3K56ac in *Sirt6^+/+^* and *Sirt6^-/-^* testes were detected by western blot. 8-week mice, n=3. (**F**) A functional diagram of Sirt6 in spermatogenesis.

In vitro screening of acetylated histone tail peptides revealed that the deacetylation activity of SIRT6 could increase acetylation of H3K9, and Sirt6-deficent cells have been shown to contain hyperacetylated H3K9 at telomeres [40]. In addition to H3K9, other lysines such as H3K18 and H3K56 are also reported to be substrates of SIRT6 [19, 41], so we tested whether Sirt6 regulates histone acetylation of these sites during spermatogenesis. H3K9ac was localized in round spermatids and elongated spermatids (Figure 6B), but no differences in H3K9ac levels were found between *Sirt6^+/+^* and *Sirt6^-/-^* testes (Figure 6C). Western blotting also confirmed that H3K9ac levels showed no significant differences between *Sirt6^+/+^* and *Sirt6^-/-^* testes (Figure 6A). As for other SIRT6 deacetylation targets, H3K18ac was localized in elongated spermatids in testes (Supplementary Figure 1), but the levels of H3K18ac in *Sirt6^-/-^* testes were similar to that of the *Sirt6^+/+^* mice (Figure 6D). Similar to H3K9ac and H3K18ac levels, no difference was found between the H3K56ac levels of *Sirt6^+/+^* and *Sirt6^-/-^* testes (Figure 6E). These results suggest that deacetylation activities of SIRT6 on H3K9ac, H3K18ac and H3K56c are not required for spermatogenesis.

## DISCUSSION

Loss of Sirt6 in C57BL mice results in accelerated aging and premature death within a month of their birth [31], whereas SIRT6-deficient monkeys die within hours after birth [42], suggesting that Sirt6 deficiency may result in deleterious outcomes that vary based on species or genetic background. In female or male Sirt6-deficient mice, Trp53 haploinsufficiency has been reported to dramatically extend mouse lifespan to several months [43]. In addition, Sirt6 global knockout in mice with a C57BL6/129svJ mixed background, but not a C57Bl/6 background, were able to survive up to one year [44]. In the present study, we used mice with a C57BL6/ICR mixed background to acquire healthier adult male mice, thus providing an opportunity to study their reproduction.

Recently, Sirt6 has been reported to participate in oocyte meiosis by modulating the acetylation status of histone H4K16 [23]. More recent studies reported that reduced expression of SIRT6 protein is closely related to dysfunctional telomeres and apoptosis in aged female mice [45]. Since the relationship between Sirt6 deficiency and premature aging has been well-established [31, 42], the spermiogenesis defect of Sirt6-knockout mice might arise from premature aging. Aging has a significant impact on male fertility, and older men exhibit notable disturbances in the reproductive process. Morphological and functional alterations in the aging testis may result in decreased hormone production, including gonadotropin-releasing hormone, luteinizing hormone, follicle-stimulating hormone and testosterone [46]. Thus, SIRT6 might protect against aging-associated pathologies since Sirt6 deletion can accelerate aging in mice.

In addition to aging, the relationship between Sirt6 deficiency and the DNA damage response has also been thoroughly investigated [41, 47, 48]. During spermatogenesis, DSB production is programmed in spermatocytes at the leptotene stage and those DSBs are eliminated by meiotic recombination [49, 50]. Because most of the meiotic recombination-related machineries share the same mechanism with somatic cell homologue recombination, it is speculated that meiotic recombination originates from mitotic homologue recombination [51]. Therefore, we had speculated that Sirt6 might participate in meiotic recombination during meiosis. To our surprise, in the current study, meiotic recombination defects were noticeably lacking in the Sirt6-knockout mice (Figure 2). These results suggest that the functional role of SIRT6 in germ cells could be considered completely different from its functional role in somatic cells.

Indeed, we found that the Sirt6-deficient spermatids arrest at the elongated spermatid stage (Figure 3). Because SIRT6 is expressed in round to elongated spermatids (Figure 1A), we believe Sirt6 should play a direct role in germ cell development. Of course, because we used a conventional *Sirt6-*knockout model, we cannot rule out the possibility of secondary effects from other tissues or organs, such as the well-known hypothalamic–pituitary axis. Indeed, we found that serum testosterone levels of 8-week *Sirt6*-knockout mice (0.14±0.08 ng/ml) were significantly lower than those of the control group (0.31±0.14 ng/ml; p=0.024). To further dissect the functional role of SIRT6, studies will need to develop tissue-specific Sirt6-knockout mouse models, such as germ cell-specific, Sertoli cell- specific and Leydig cell-specific Sirt6 knock out models. Given that SIRT6 is an enzyme with NAD^+^-dependent deacetylase activity and mono-ADP-ribosyl transferase activity that has multiple substrates [52–55], and neither of the tested deacetylation related substrates is required during spermatogenesis, the mono-ADP-ribosylation activity of SIRT6 might be very important to this process. Thus, SIRT6’s effect on spermatogenesis may result from one or more of the enzyme’s known substrates, or totally unknown substrates that might be specific for germ-cell development or even independent of SIRT6’s enzyme activities at all (Figure 6F). All these open questions still need further investigation in the near future.

Our current study provides evidence and details on the functional role of SIRT6 in spermatogenesis. Overall, we found that Sirt6 global knockout resulted in infertility in male mice, and spermatogenesis in *Sirt6^-/-^* mice was arrested at the elongated spermatid stage. Furthermore, loss of Sirt6 in mice resulted in an elevated number of apoptotic spermatids. Therefore, our findings establish a novel link between Sirt6 and male fertility and suggest an essential role of Sirt6 in spermatogenesis.

## MATERIALS AND METHODS

### Animals

Mice were maintained in a 12:12 light/ dark cycle with food and water available *ad libitum* in cages held at 23±2°C. *Sirt6* global knockout (*Sirt6^-/-^*) mice were generated using Sirt6-floxed (*Sirt6^flox/flox^*) mice on a C57BL6/ICR mixed background. Mice harboring a floxed conditional knockout cassette of C57BL6 *Sirt6* were purchased from EUCOMM. Mice carrying the floxed *Sirt6* allele (*Sirt6^+/flox^*) were mated to mice expressing Cre recombinase under control of the ZP3 promoter (Zp3-Cre) [56]. To generate heterozygous *Sirt6*-knockout (*Sirt6^+/−^*) mice, these *Sirt6^+/flox^*; Zp3-Cre or *Sirt6^flox /flox^*; Zp3-Cre mice were further mated with WT female mice. Zp3-Cre mice were purchased from Jackson Laboratory. WT ICR mice were purchased from SPF (Beijing) Biotechnology, LLC.

All the 8-week male mice were sacrificed by cervical dislocation before testes collection. All the animal experiments were approved by the Animal Research Panel of the Committee on Research Practice of the University of the Chinese Academy of Sciences (Approval number: IOZ20150041).

### Tissue collection and histological analysis

For histological examination, at least three adult mice for each genotype were analyzed. The testes were dissected and fixed with Bouin’s fixative for up to 24 h at 4 °C. Next, the testes were dehydrated using graded ethanol and embedded in paraffin. 5μm sections were obtained and transferred to glass slides. After deparaffinization, sections were stained with H&E or stained with PAS-hematoxylin to determine the seminiferous epithelia cycle stages.

### Chromosome spreads of spermatocyte

Chromosome spreads of spermatocytes were performed using a drying-down technique as previously described [57]. Briefly, the testes were dissected, and the tubules were washed with phosphate-buffered saline (PBS), pH 7.4 at room temperature. Next, the tubules were placed in a hypotonic extraction buffer containing 30 mM Tris, 50 mM sucrose, 17 mM trisodium citrate dihydrate, 5 mM EDTA, 0.5 mM DTT and 0.5 mM phenylmethylsulfonyl fluoride (PMSF), pH 8.2, for 30 min at room temperature. Subsequently, the tubules were homogenized in 100 mM sucrose, pH 8.2, on a clean glass slide and were pipetted repeatedly to make a suspension. The cell suspensions were placed on slides pre-coated with 1% paraformaldehyde (PFA) (Solarbio), pH 9.2, and 0.15% Triton X-100. The slides were dried for at least 2 h in a closed box with high humidity in room temperature. Finally, the slides were washed twice with PBS for 10 min and stained with antibodies according to standard protocols.

### Antibodies

Rabbit anti-SIRT6 polyclonal antibody was purchased from sigma (S4197) for western blotting and Abcam(ab62739) for immunofluorescence staining. Mouse anti-SYCP3 (150292) and rabbit anti-SYCP3 (ab15093) were purchased from Abcam (Cambridge, MA). Rabbit anti-SYCP1 (NB300-228c) was purchased from Novus Biologicals (Littleton, CO). Mouse anti-γH2AX (05-636) was purchased from Merck Millipore (Darmstadt, Germany). Rabbit anti-H2A (ab18975) was purchased from Abcam (Cambridge, MA). Rabbit anti-H3K9ac(P30050M) and mouse anti-ACTIN antibody (M20011L) were purchased from Abmart, H3K18ac and H3K56ac antibody kits (9927T) were purchased from Cell Signaling Technology. Alexa Fluor 488 -lectin PNA was purchased from Molecular Probes. Goat FITC-conjugated secondary antibodies for rabbit, goat FITC-conjugated secondary antibodies for mouse, goat TRITC conjugated secondary antibodies for rabbit, and goat TRITC conjugated secondary antibodies for mouse were purchased from Zhong Shan Jin Qiao (Beijing, China). Alexa Fluor 680-conjugated goat anti-mouse and Alexa Fluor 800-conjugated goat anti-rabbit secondary antibodies, used in western blot analysis, were purchased from Invitrogen.

### Immunoblotting

Tissue extracts were prepared in cold RIPA buffer (25 mM Tris–HCl, pH 7.6, 350 mM NaCl, 1% Nonidet P-40, 1% sodium deoxycholate, and 0.1% sodium dodecyl sulfate) supplemented with 1 mM PMSF and a protein inhibitor cocktail (Roche Diagnostics, 04693116001, Rotkreuz, Switzer-land) using a Dounce homogenizer. After a brief sonication, the cell lysates were incubated on ice for 30 min. The samples were centrifuged at ~14 000 ×g for 15 min at 4 °C to pellet the cell debris, and the supernatant was transferred to a new tube for further analysis. Protein lysates were separated via SDS-PAGE and electro-transferred to nitrocellulose membranes. After incubation with respective primary at 4 °C overnight and secondary antibodies in room temperature one hour, the membrane was scanned using an ODYSSEY Sa Infrared Imaging System (LI-COR Biosciences, Lincoln, NE, USA).

### Immunofluorescence and TUNEL assay

Testes were collected and immediately embedded in OCT compound (Tissue-Tek) and cut into 7μm sections using a microtome-cryostat (CM1950, Leica). Sections were fixed in 4% PFA for 10 min and then rinsed three times with PBS at room temperature (pH7.4), treated with 0.1% Triton X-100 for 10 min and rinsed three times with PBS. The samples were then blocked in 5% bovine serum albumin (BSA) in PBS for 30 min, and incubated with the respective primary antibody at 4°C overnight. After rinsing three times with PBS, the sections were incubated with a FITC-conjugated secondary antibody at 1:200 for 1 h at 37°C. DAPI (D3571, Life Technologies) was used to label nuclei. Images were captured immediately using a TCS SP8 microscope (Leica, Wetzlar, Germany). To examine chromosome immunofluorescence, the chromosome spreads of spermatocytes were rinsed with PBS three times and blocked with 5% BSA, followed by staining as detailed above. To detect apoptotic cells in the testis, we used the terminal deoxynucleotidyl transferase dUTP nick end-labeling (TUNEL) assay kit (In Situ Cell Death Detection Kit; Roche, 11684795910) [58]. Briefly, sections of the testes were deparaffinized and boiled for 15 min in sodium citrate buffer for antigen retrieval. Next, the slides were treated with H_2_O_2_ for 10 min at room temperature and sodium citrate for 2 min on ice. The slides were rinsed twice with PBS, and the TUNEL reaction mixture was added and incubated in a humidified atmosphere for 60 min at 37 °C in the dark, followed by staining as described above.

### Hormone measurement

Serum levels of mouse testosterone were measured using a radioimmunoassay kit (Beijing Sinouk Institute of Biological Technology) as previously described [59].

### Statistical analysis

All experiments were repeated at least three times and the data are presented as the mean ±SEM. In each group, we used *Sirt6^+/+^* littermates as negative controls. Statistical analyses were conducted using GraphPad PRISM version 8.02. The statistical significance of the differences between the mean values was measured by a Student’s t-test with a paired two-tailed distribution. The data were considered significant when the P-value was less than 0.05 (*), 0.01 (**) or 0.001(***).

### Ethical approval

All of the animal experiments were performed according to approved institutional animal care and use committee (IACUC) protocols (#08-133) of the Institute of Zoology, Chinese Academy of Sciences. All surgery was performed under sodium pentobarbital anesthesia, and every effort was made to minimize suffering.

## Supplementary Material

Supplementary Figure 1

## References

[1] Hess RA, Renato de Franca L. Spermatogenesis and cycle of the seminiferous epithelium. Adv Exp Med Biol. 2008; 636:1–15. 10.1007/978-0-387-09597-4_1 19856159

[2] Page SL, Hawley RS. Chromosome choreography: the meiotic ballet. Science. 2003; 301:785–89. 10.1126/science.1086605 12907787

[3] Parvinen M. Regulation of the seminiferous epithelium. Endocr Rev. 1982; 3:404–17. 10.1210/edrv-3-4-404 6295753

[4] Hermo L, Pelletier RM, Cyr DG, Smith CE. Surfing the wave, cycle, life history, and genes/proteins expressed by testicular germ cells. Part 1: background to spermatogenesis, spermatogonia, and spermatocytes. Microsc Res Tech. 2010; 73:241–78. 10.1002/jemt.20783 19941293

[5] Calvel P, Rolland AD, Jégou B, Pineau C. Testicular postgenomics: targeting the regulation of spermatogenesis. Philos Trans R Soc Lond B Biol Sci. 2010; 365:1481–500. 10.1098/rstb.2009.0294 20403865 PMC2871924

[6] Imai S, Armstrong CM, Kaeberlein M, Guarente L. Transcriptional silencing and longevity protein Sir2 is an NAD-dependent histone deacetylase. Nature. 2000; 403:795–800. 10.1038/35001622 10693811

[7] North BJ, Verdin E. Sirtuins: Sir2-related NAD-dependent protein deacetylases. Genome Biol. 2004; 5:224. 10.1186/gb-2004-5-5-224 15128440 PMC416462

[8] Blander G, Guarente L. The Sir2 family of protein deacetylases. Annu Rev Biochem. 2004; 73:417–35. 10.1146/annurev.biochem.73.011303.073651 15189148

[9] Frye RA. Phylogenetic classification of prokaryotic and eukaryotic Sir2-like proteins. Biochem Biophys Res Commun. 2000; 273:793–98. 10.1006/bbrc.2000.3000 10873683

[10] Michishita E, Park JY, Burneskis JM, Barrett JC, Horikawa I. Evolutionarily conserved and nonconserved cellular localizations and functions of human SIRT proteins. Mol Biol Cell. 2005; 16:4623–35. 10.1091/mbc.e05-01-0033 16079181 PMC1237069

[11] Frye RA. Characterization of five human cDNAs with homology to the yeast SIR2 gene: Sir2-like proteins (sirtuins) metabolize NAD and may have protein ADP-ribosyltransferase activity. Biochem Biophys Res Commun. 1999; 260:273–79. 10.1006/bbrc.1999.0897 10381378

[12] North BJ, Marshall BL, Borra MT, Denu JM, Verdin E. The human Sir2 ortholog, SIRT2, is an NAD+-dependent tubulin deacetylase. Mol Cell. 2003; 11:437–44. 10.1016/s1097-2765(03)00038-8 12620231

[13] Shi T, Wang F, Stieren E, Tong Q. SIRT3, a mitochondrial sirtuin deacetylase, regulates mitochondrial function and thermogenesis in brown adipocytes. J Biol Chem. 2005; 280:13560–67. 10.1074/jbc.M414670200 15653680

[14] Grabowska W, Sikora E, Bielak-Zmijewska A. Sirtuins, a promising target in slowing down the ageing process. Biogerontology. 2017; 18:447–76. 10.1007/s10522-017-9685-9 28258519 PMC5514220

[15] Vachharajani VT, Liu T, Wang X, Hoth JJ, Yoza BK, McCall CE. Sirtuins link inflammation and metabolism. J Immunol Res. 2016; 2016:8167273. 10.1155/2016/8167273 26904696 PMC4745579

[16] McBurney MW, Yang X, Jardine K, Hixon M, Boekelheide K, Webb JR, Lansdorp PM, Lemieux M. The mammalian SIR2alpha protein has a role in embryogenesis and gametogenesis. Mol Cell Biol. 2003; 23:38–54. 10.1128/mcb.23.1.38-54.2003 12482959 PMC140671

[17] Tatone C, Di Emidio G, Barbonetti A, Carta G, Luciano AM, Falone S, Amicarelli F. Sirtuins in gamete biology and reproductive physiology: emerging roles and therapeutic potential in female and male infertility. Hum Reprod Update. 2018; 24:267–89. 10.1093/humupd/dmy003 29447380

[18] Zhou XL, Xu JJ, Ni YH, Chen XC, Zhang HX, Zhang XM, Liu WJ, Luo LL, Fu YC. SIRT1 activator (SRT1720) improves the follicle reserve and prolongs the ovarian lifespan of diet-induced obesity in female mice via activating SIRT1 and suppressing mTOR signaling. J Ovarian Res. 2014; 7:97. 10.1186/s13048-014-0097-z 25330910 PMC4232623

[19] Cinco R, Digman MA, Gratton E, Luderer U. Spatial characterization of bioenergetics and metabolism of primordial to preovulatory follicles in whole ex vivo murine ovary. Biol Reprod. 2016; 95:129. 10.1095/biolreprod.116.142141 27683265 PMC5315427

[20] Liu M, Yin Y, Ye X, Zeng M, Zhao Q, Keefe DL, Liu L. Resveratrol protects against age-associated infertility in mice. Hum Reprod. 2013; 28:707–17. 10.1093/humrep/des437 23293221

[21] Zhang L, Hou X, Ma R, Moley K, Schedl T, Wang Q. Sirt2 functions in spindle organization and chromosome alignment in mouse oocyte meiosis. FASEB J. 2014; 28:1435–45. 10.1096/fj.13-244111 24334550 PMC3929683

[22] Zhang L, Han L, Ma R, Hou X, Yu Y, Sun S, Xu Y, Schedl T, Moley KH, Wang Q. Sirt3 prevents maternal obesity-associated oxidative stress and meiotic defects in mouse oocytes. Cell Cycle. 2015; 14:2959–68. 10.1080/15384101.2015.1026517 25790176 PMC4825572

[23] Han L, Ge J, Zhang L, Ma R, Hou X, Li B, Moley K, Wang Q. Sirt6 depletion causes spindle defects and chromosome misalignment during meiosis of mouse oocyte. Sci Rep. 2015; 5:15366. 10.1038/srep15366 26481302 PMC4612726

[24] Zhang T, Zhou Y, Li L, Wang HH, Ma XS, Qian WP, Shen W, Schatten H, Sun QY. SIRT1, 2, 3 protect mouse oocytes from postovulatory aging. Aging (Albany NY). 2016; 8:685–96. 10.18632/aging.100911 26974211 PMC4925822

[25] Kolthur-Seetharam U, Teerds K, de Rooij DG, Wendling O, McBurney M, Sassone-Corsi P, Davidson I. The histone deacetylase SIRT1 controls male fertility in mice through regulation of hypothalamic-pituitary gonadotropin signaling. Biol Reprod. 2009; 80:384–91. 10.1095/biolreprod.108.070193 18987333

[26] Liu C, Song Z, Wang L, Yu H, Liu W, Shang Y, Xu Z, Zhao H, Gao F, Wen J, Zhao L, Gui Y, Jiao J, et al. Sirt1 regulates acrosome biogenesis by modulating autophagic flux during spermiogenesis in mice. Development. 2017; 144:441–51. 10.1242/dev.147074 28003215

[27] Coussens M, Maresh JG, Yanagimachi R, Maeda G, Allsopp R. Sirt1 deficiency attenuates spermatogenesis and germ cell function. PLoS One. 2008; 3:e1571. 10.1371/journal.pone.0001571 18270565 PMC2216432

[28] Kanfi Y, Naiman S, Amir G, Peshti V, Zinman G, Nahum L, Bar-Joseph Z, Cohen HY. The sirtuin SIRT6 regulates lifespan in male mice. Nature. 2012; 483:218–21. 10.1038/nature10815 22367546

[29] Zhong L, D’Urso A, Toiber D, Sebastian C, Henry RE, Vadysirisack DD, Guimaraes A, Marinelli B, Wikstrom JD, Nir T, Clish CB, Vaitheesvaran B, Iliopoulos O, et al. The histone deacetylase Sirt6 regulates glucose homeostasis via Hif1alpha. Cell. 2010; 140:280–93. 10.1016/j.cell.2009.12.041 20141841 PMC2821045

[30] Kugel S, Sebastián C, Fitamant J, Ross KN, Saha SK, Jain E, Gladden A, Arora KS, Kato Y, Rivera MN, Ramaswamy S, Sadreyev RI, Goren A, et al. SIRT6 suppresses pancreatic cancer through control of Lin28b. Cell. 2016; 165:1401–15. 10.1016/j.cell.2016.04.033 27180906 PMC4892983

[31] Mostoslavsky R, Chua KF, Lombard DB, Pang WW, Fischer MR, Gellon L, Liu P, Mostoslavsky G, Franco S, Murphy MM, Mills KD, Patel P, Hsu JT, et al. Genomic instability and aging-like phenotype in the absence of mammalian SIRT6. Cell. 2006; 124:315–29. 10.1016/j.cell.2005.11.044 16439206

[32] Palmer NO, Fullston T, Mitchell M, Setchell BP, Lane M. SIRT6 in mouse spermatogenesis is modulated by diet-induced obesity. Reprod Fertil Dev. 2011; 23:929–39. 10.1071/RD10326 21871212

[33] Zickler D, Kleckner N. Meiotic chromosomes: integrating structure and function. Annu Rev Genet. 1999; 33:603–754. 10.1146/annurev.genet.33.1.603 10690419

[34] Ward IM, Chen J. Histone H2AX is phosphorylated in an ATR-dependent manner in response to replicational stress. J Biol Chem. 2001; 276:47759–62. 10.1074/jbc.C100569200 11673449

[35] Meuwissen RL, Offenberg HH, Dietrich AJ, Riesewijk A, van Iersel M, Heyting C. A coiled-coil related protein specific for synapsed regions of meiotic prophase chromosomes. EMBO J. 1992; 11:5091–100. 1464329 10.1002/j.1460-2075.1992.tb05616.x PMC556987

[36] Sainio-Pöllänen S, Henriksén K, Parvinen M, Simell O, Pöllänen P. Stage-specific degeneration of germ cells in the seminiferous tubules of non-obese diabetic mice. Int J Androl. 1997; 20:243–53. 10.1046/j.1365-2605.1997.00061.x 9401828

[37] Braun RE. Packaging paternal chromosomes with protamine. Nat Genet. 2001; 28:10–12. 10.1038/ng0501-10 11326265

[38] Berruti G, Paiardi C. Acrosome biogenesis: revisiting old questions to yield new insights. Spermatogenesis. 2011; 1:95–98. 10.4161/spmg.1.2.16820 22319656 PMC3271650

[39] Kang-Decker N, Mantchev GT, Juneja SC, McNiven MA, van Deursen JM. Lack of acrosome formation in hrb-deficient mice. Science. 2001; 294:1531–33. 10.1126/science.1063665 11711676

[40] Michishita E, McCord RA, Berber E, Kioi M, Padilla-Nash H, Damian M, Cheung P, Kusumoto R, Kawahara TL, Barrett JC, Chang HY, Bohr VA, Ried T, et al. SIRT6 is a histone H3 lysine 9 deacetylase that modulates telomeric chromatin. Nature. 2008; 452:492–96. 10.1038/nature06736 18337721 PMC2646112

[41] Toiber D, Erdel F, Bouazoune K, Silberman DM, Zhong L, Mulligan P, Sebastian C, Cosentino C, Martinez-Pastor B, Giacosa S, D’Urso A, Näär AM, Kingston R, et al. SIRT6 recruits SNF2H to DNA break sites, preventing genomic instability through chromatin remodeling. Mol Cell. 2013; 51:454–68. 10.1016/j.molcel.2013.06.018 23911928 PMC3761390

[42] Zhang W, Wan H, Feng G, Qu J, Wang J, Jing Y, Ren R, Liu Z, Zhang L, Chen Z, Wang S, Zhao Y, Wang Z, et al. SIRT6 deficiency results in developmental retardation in cynomolgus monkeys. Nature. 2018; 560:661–65. 10.1038/s41586-018-0437-z 30135584

[43] Ghosh S, Wong SK, Jiang Z, Liu B, Wang Y, Hao Q, Gorbunova V, Liu X, Zhou Z. Haploinsufficiency of Trp53 dramatically extends the lifespan of Sirt6-deficient mice. Elife. 2018; 7:e32127. 10.7554/eLife.32127 29474172 PMC5825207

[44] Huang W, Liu H, Zhu S, Woodson M, Liu R, Tilton RG, Miller JD, Zhang W. Sirt6 deficiency results in progression of glomerular injury in the kidney. Aging (Albany NY). 2017; 9:1069–83. 10.18632/aging.101214 28351995 PMC5391219

[45] Ge J, Li C, Li C, Huang Z, Zeng J, Han L, Wang Q. SIRT6 participates in the quality control of aged oocytes via modulating telomere function. Aging (Albany NY). 2019; 11:1965–76. 10.18632/aging.101885 30926765 PMC6503879

[46] Almeida S, Rato L, Sousa M, Alves MG, Oliveira PF. Fertility and sperm quality in the aging male. Curr Pharm Des. 2017; 23:4429–37. 10.2174/1381612823666170503150313 28472913

[47] Kaidi A, Weinert BT, Choudhary C, Jackson SP. Human SIRT6 promotes DNA end resection through CtIP deacetylation. Science. 2010; 329:1348–53. 10.1126/science.1192049 20829486 PMC3276839

[48] Rizzo A, Iachettini S, Salvati E, Zizza P, Maresca C, D'Angelo C, Benarroch-Popivker D, Capolupo A, Del Gaudio F, Cosconati S, Di Maro S, Merlino F, Novellino E, et al SIRT6 interacts with TRF2 and promotes its degradation in response to DNA damage. Nucleic acids research. 2017; 45:1820–1834. 27923994 10.1093/nar/gkw1202 PMC5389694

[49] Keeney S, Giroux CN, Kleckner N. Meiosis-specific DNA double-strand breaks are catalyzed by Spo11, a member of a widely conserved protein family. Cell. 1997; 88:375–84. 10.1016/s0092-8674(00)81876-0 9039264

[50] Blanco-Rodríguez J. Programmed phosphorylation of histone H2AX precedes a phase of DNA double-strand break-independent synapsis in mouse meiosis. Reproduction. 2012; 144:699–712. 10.1530/REP-12-0326 23035256

[51] Ciccia A, Elledge SJ. The DNA damage response: making it safe to play with knives. Mol Cell. 2010; 40:179–204. 10.1016/j.molcel.2010.09.019 20965415 PMC2988877

[52] Rezazadeh S, Yang D, Tombline G, Simon M, Regan SP, Seluanov A, Gorbunova V. SIRT6 promotes transcription of a subset of NRF2 targets by mono-ADP-ribosylating BAF170. Nucleic Acids Res. 2019; 47:7914–28. 10.1093/nar/gkz528 31216030 PMC6736037

[53] Mao Z, Hine C, Tian X, Van Meter M, Au M, Vaidya A, Seluanov A, Gorbunova V. SIRT6 promotes DNA repair under stress by activating PARP1. Science. 2011; 332:1443–46. 10.1126/science.1202723 21680843 PMC5472447

[54] Van Meter M, Kashyap M, Rezazadeh S, Geneva AJ, Morello TD, Seluanov A, Gorbunova V. SIRT6 represses LINE1 retrotransposons by ribosylating KAP1 but this repression fails with stress and age. Nat Commun. 2014; 5:5011. 10.1038/ncomms6011 25247314 PMC4185372

[55] Liszt G, Ford E, Kurtev M, Guarente L. Mouse Sir2 homolog SIRT6 is a nuclear ADP-ribosyltransferase. J Biol Chem. 2005; 280:21313–20. 10.1074/jbc.M413296200 15795229

[56] Hammond SS, Matin A. Tools for the genetic analysis of germ cells. Genesis. 2009; 47:617–27. 10.1002/dvg.20539 19548313

[57] Peters AH, Plug AW, van Vugt MJ, de Boer P. A drying-down technique for the spreading of mammalian meiocytes from the male and female germline. Chromosome Res. 1997; 5:66–68. 10.1023/a:1018445520117 9088645

[58] Song ZH, Yu HY, Wang P, Mao GK, Liu WX, Li MN, Wang HN, Shang YL, Liu C, Xu ZL, Sun QY, Li W. Germ cell-specific Atg7 knockout results in primary ovarian insufficiency in female mice. Cell Death Dis. 2015; 6:e1589. 10.1038/cddis.2014.559 25590799 PMC4669757

[59] Mason-Garcia M, Vigh S, Comaru-Schally AM, Redding TW, Somogyvari-Vigh A, Horvath J, Schally AV. Radioimmunoassay for 6-D-tryptophan analog of luteinizing hormone-releasing hormone: measurement of serum levels after administration of long-acting microcapsule formulations. Proc Natl Acad Sci USA. 1985; 82:1547–51. 10.1073/pnas.82.5.1547 3156381 PMC397301

