# Supplementary material for: Sirt6 is required for spermatogenesis in mice
Source: Aging (Albany NY). 2020 Sep 11;12(17):17099–113. doi: 10.18632/aging.103641 (PMC7521524; doi:10.18632/aging.103641)
Supplement: Supplementary Figure 1 [file aging-12-103641-s001..pdf]

SUPPLEMENTARY FIGURE

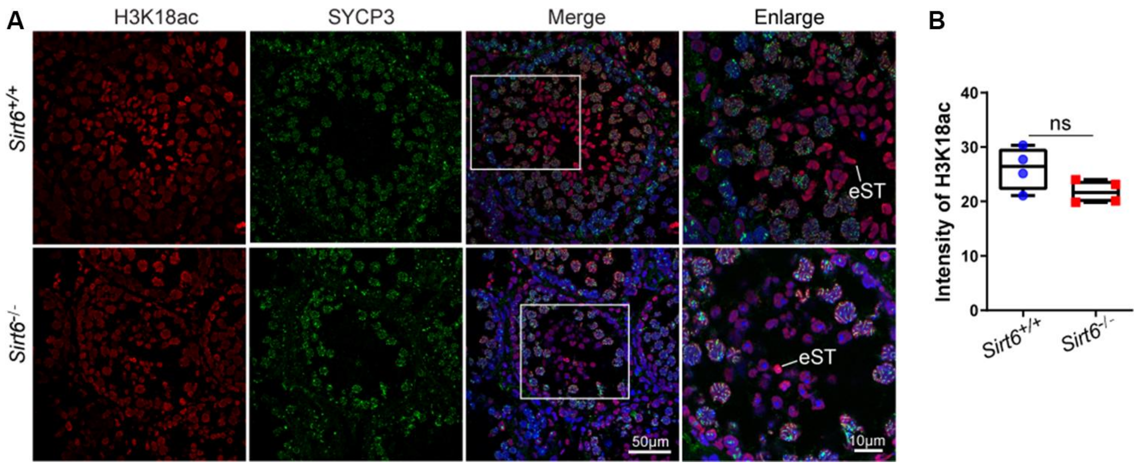

Supplementary **Figure 1**. **The role of Sirt6 in spermatogenesis is independent of H3K18ac.** (**A**) Localization of H3K18ac in *Sirt6*$^{+/+}$ and *Sirt6*$^{-/-}$ seminiferous tubules. Testes sections of *Sirt6*$^{+/+}$ and *Sirt6*$^{-/-}$ stained with SYCP3 (green) and H3K18ac (red) antibodies. 8-week mice, n=4. (**B**) Quantification of H3K18ac intensity of elongated spermatids in the *Sirt6*$^{+/+}$ and *Sirt6*$^{-/-}$ mice. *Sirt6*$^{+/+}$, 26.09±3.95; *Sirt6*$^{-/-}$, 21.79±2.12. 8-week mice, n=4; 200 cells were used for each group. Data are presented as mean ± SEM.
